# The Impact of Nasal Pathologies on the Success of External Dacryocystorhinostomy

**DOI:** 10.7759/cureus.90230

**Published:** 2025-08-16

**Authors:** Suzan Dogruya, Ömer Can Kayikcioglu, Özcan Rasim Kayikcioglu

**Affiliations:** 1 Ophthalmology, Uşak University, Uşak, TUR; 2 Ophthalmology, Başkent University, İzmir, TUR; 3 Ophthalmology, Kayikcioglu Ophthalmology Clinic, Manisa, TUR

**Keywords:** bi-canalicular silicone intubation, external dacryocystorhinostomy, nasal pathologies, nasolacrimal duct obstruction, paranasal sinus computed tomography

## Abstract

Background

External dacryocystorhinostomy (Ex-DCR) is a widely used surgical procedure for nasolacrimal duct obstruction, but its success may be influenced by coexisting nasal pathologies. Preoperative evaluation with paranasal sinus computed tomography (CT) can assist in surgical planning and decision-making.

Purpose

To identify nasal pathologies in patients who underwent Ex-DCR with bi-canalicular silicone tube intubation for dacryostenosis by reviewing their paranasal sinus CT scans and to evaluate the impact of these pathologies on surgical success.

Method

We retrospectively analyzed 78 patients with paranasal sinus CT. Nine patients underwent bilateral operations. Patients with primary acquired chronic dacryocystitis (PACD) and revision external dacryocystorhinostomy (rE-DCR) were included in the study. All patients underwent Ex-DCR with silicone tube intubation. Anatomic success was defined as the patency of the lacrimal passage after the removal of silicone tube six months after tube intubation. The study investigated septal deviation, concha bullosa, inferior concha hypertrophy, and sinus inflammation.

Results

The mean age of the patients was 58.39±11.67 years, with 11 men (14.1%) and 67 women (85.9%). Eighty (92%) patients had PACD and seven (8%) patients had rE-DCR. The mean follow-up period was 2.45±2.14 years. Among these, no pathology was detected in 26 patients (29.9%). Fifty-five (90%) of 61 patients with nasal pathology had anatomical success, but in six patients, no success was achieved. There was no statistically significant difference in anatomical success between patients with and without nasal pathologies (p>0.05). There was no significant difference in anatomical success between patients with PACD and rE-DCR (p=0.09).

Conclusion

Ex-DCR with bi-canalicular silicone intubation remains a safe and effective treatment for nasolacrimal duct obstruction, even in the presence of common nasal pathologies. While preoperative CT can be informative, these structural nasal findings do not appear to compromise surgical success in most cases.

## Introduction

External dacryocystorhinostomy (Ex-DCR) is still considered to have a high success rate for the treatment of distal lacrimal duct obstruction [1]. Ex-DCR was first reported by Addeo Toti in 1904 [2]. Later in 1921, Dupy-Dutemps and Bourguet described the suturing of mucosal flaps to form an anastomosis [3]. Ohm proposed suturing anterior and posterior flaps of nasal mucosa to the lacrimal sac in 1926 [4]. Older performed silicone tube application in Ex-DCR [5]. DCR involves creating an osteotomy and opening the lacrimal sac into the nasal cavity, establishing a functional pathway from the canaliculi to the nose. This procedure can be performed using either an external or endonasal approach [6]. Studies show that silicone intubation improves success rates of Ex-DCR, ranging from 80% to 95% [7]. In endoscopic DCR, this rate is between 73% and 95% [8,9].

While primary acquired nasolacrimal duct (NLD) obstruction is primarily seen as an ophthalmological issue, rhinological factors play a significant role in its etiology [10]. Due to the anatomical proximity of the lateral nasal wall to the lacrimal system, diseases in this region may contribute to primary NLD. Some studies have reported that NLD obstruction is more common in individuals with deviated septum than in those without [11]. Agger nasi cells and maxillary sinusitis may also affect the incidence of obstruction [12]. Conditions related to nasal structures like septal deviation, nasal polyps, chronic sinusitis, and hypertrophic turbinates can negatively affect DCR outcomes by influencing postoperative healing. These pathologies can also promote inflammatory processes or accelerate scar tissue formation, leading to obstruction of the anastomosis site [10].

To the best of our knowledge, there are limited studies evaluating the association between CT-detected nasal pathologies and anatomical success of Ex-DCR, especially in the Turkish population [13-15].

This study aimed to evaluate the relationship between nasal pathologies identified on computed tomography and the short-term anatomical success of Ex-DCR with silicone tube intubation in patients with nasolacrimal duct obstruction.

## Materials and methods

In this study, 87 surgeries of 78 patients who underwent Ex-DCR and silicone tube intubation for NLD obstruction between 2018 and 2023 at Uşak Training and Research Hospital were retrospectively analyzed. The demographic data and medical histories of the patients were thoroughly examined. A comprehensive eye examination, including visual acuity, and biomicroscopic evaluation was performed. Lacrimal irrigation was performed under topical anesthesia in all patients. Nasolacrimal duct obstruction was considered if saline did not reach the nose during irrigation of the system during rigid probing with a cannula. Tear drainage was evaluated by dacryoscintigraphy.

All patients underwent a preoperative otolaryngologic consultation to evaluate potential nasal pathologies. The otolaryngologist performed a comprehensive clinical examination focused on identifying concha abnormalities, sinusitis, and nasal septal deviation. When clinically indicated, paranasal sinus computed tomography (CT) scans were ordered to further assess anatomical structures.

Preoperative CT scans were systematically reviewed by an experienced otolaryngologist. The assessment focused on the presence and severity of nasal septal deviation, concha bullosa, inferior turbinate hypertrophy, and radiological signs of sinus inflammation. Septal deviation was classified based on the angle and extent of deviation from the midline. Concha bullosa was defined as pneumatization of the middle turbinate, while inferior turbinate hypertrophy was evaluated by comparing turbinate size relative to the nasal cavity. Sinus inflammation was determined by the presence of mucosal thickening, air-fluid levels, or sinus opacification. All CT findings were documented and analyzed in relation to surgical outcomes.

Patients presenting with epiphora, failed lacrimal irrigation, and distal lacrimal drainage system obstruction demonstrated by dacryoscintigraphy were included in the study. All patients underwent external dacryocystorhinostomy (Ex-DCR) with bicanalicular silicone tube intubation. Patients who had undergone endoscopic DCR or those who received Ex-DCR without silicone tube intubation were excluded from the study. Both patients with primary acquired chronic dacryocystitis (PACD) and those who had previously undergone failed DCR surgery (revision cases) were included.

All Ex-DCR procedures were performed under general anesthesia. A vertical skin incision was made approximately 10 mm medial to the medial canthus. After blunt dissection, the lacrimal sac and adjacent nasal bone were exposed. A bony ostium approximately 12×10 mm in size was created using an ENT (ear, nose, and throat) TUR motor (Aesculap®, Tuttlingen, Germany). An U-shaped anterior mucosal flap was fashioned from both the lacrimal sac and nasal mucosa, and no posterior flap was created. The anterior flaps were sutured together with 6-0 Vicryl sutures to achieve mucosa-to-mucosa anastomosis. A bicanalicular silicone stent was placed in all patients. In all patients, the silicone tube was removed approximately six months after surgery. Skin closure was performed using interrupted 6-0 Prolene sutures.

Postoperative follow-up after Ex-DCR included evaluations on the first postoperative day to assess early complications such as edema, hematoma, or infection. Sutures were typically removed within the first week. Patients were monitored regularly for signs of inflammation, lacrimal drainage system patency, and silicone tube position. Silicone stents were generally removed approximately six months after surgery. Additional follow-up visits were scheduled every six months to monitor long-term surgical outcomes, symptom resolution, and to detect any late complications.

Anatomic success was defined as the patency of the lacrimal passage by irrigation one month after removal of the silicone tube. For this purpose, saline was administered through the upper or lower punctum using a lacrimal cannula. Preoperative paranasal sinus CT scans of the patients were reviewed. The presence of septal deviation, concha bullosa, inferior concha hypertrophy, and sinus inflammation was assessed. The effect of preoperative nasal pathologies on anatomical success was statistically evaluated.

Statistical analysis was performed using SPSS 22.0 software package (IBM Corp., Armonk, NY), and a P-value of less than 0.05 was considered statistically significant. Descriptive statistical methods (mean and standard deviation) were used to evaluate the data. Qualitative data were compared using the chi-square test and Fisher's exact test. The effect of nasal pathology on surgical success was evaluated by logistic regression analysis.

The study adhered to the principles of the Helsinki Declaration, and the study protocol was approved by the Ethics Committee of Uşak University (approval date: November 7, 2024/459-459-06). Written informed consent was obtained from all patients prior to their inclusion in the study.

## Results

The average age of the patients was 58.39±11.67 years, with 11 men (14.1%) and 67 women (89.9%). PACD was observed in 80 eyes (92%), while rE-DCR was found in seven eyes (8%). The mean follow-up period was 2.45±2.14 years. In all patients, silicone tubes were removed six months after the procedure.

**Figure 1:**
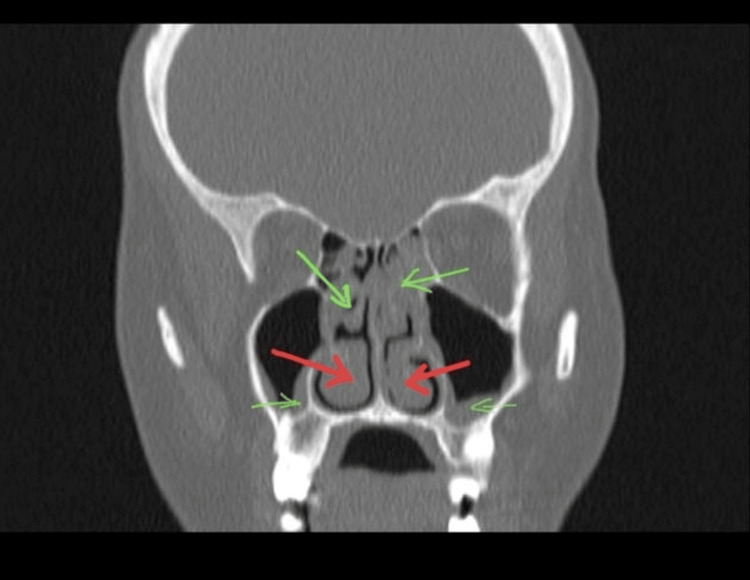
A coronal paranasal CT shows bilateral maxillary sinusitis and inferior turbinate hypertrophy The green arrows indicate mucosal thickening of the ethmoid and maxillary sinuses, which suggests sinusitis. The red arrows indicate bilateral inferior turbinate hypertrophy.

**Figure 2:**
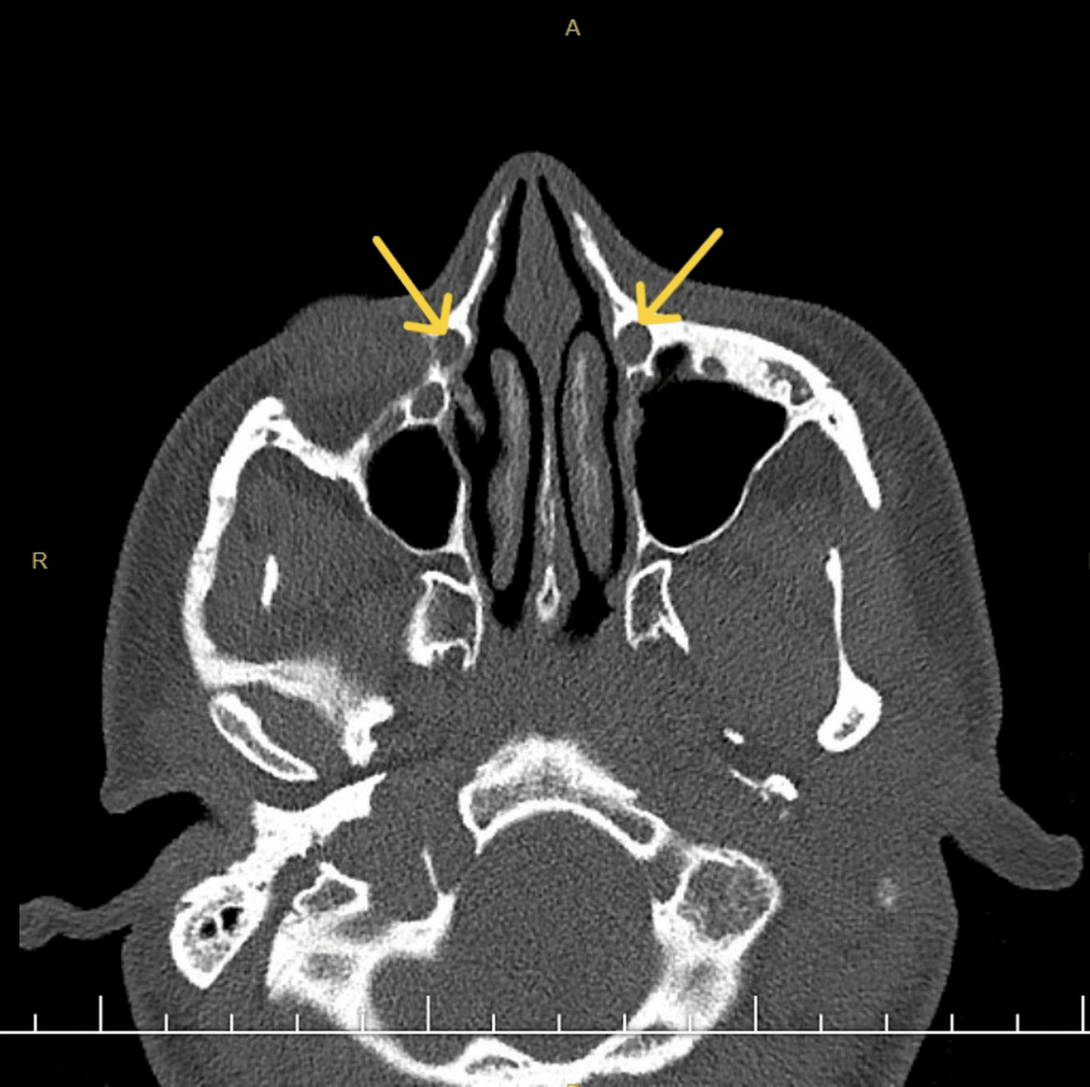
In the horizontal paranasal CT, left inferior turbinate hypertrophy are observed. Yellow arrow points to the nasolacrimal duct.

The paranasal sinus CT showed no nasal pathology in 26 patients (29.9%). However, in the remaining 61 patients (70.1%), various findings were identified, including concha abnormalities, sinus inflammation, septal deviation, and combinations of concha abnormalities with septal deviation (Figures 1, 2 and Table 1).

**Table 1:** Clinical characteristics and anatomical success rates of patients PACD: primary acquired chronic dacryocystitis, rE-DCR: revision external dacryocystorhinostomy

Variable	Total (n=87)	Anatomical Success, n (%)	Anatomical Failure, n (%)
Diagnosis			
• PACD	80 (92.0%)	75 (93.7%)	5 (6.3%)
• rE-DCR	7 (8.0%)	5 (71.4%)	2 (28.6%)
Nasal pathology			
• Present	61 (70.1%)	55 (90.2%)	6 (9.8%)
• Absent	26 (29.9%)	25 (96.2%)	1 (3.8%)
Types of failure in nasal pathology group			
• Concha hypertrophy+sinusitis	–	–	1
• Septal deviation	–	–	4
• Sinusitis alone	–	–	1
• Without nasal pathology	–	–	1

The anatomic success rate was 96.2% (25 patients) in 26 patients without nasal pathology. Among the 61 patients with nasal pathologies, anatomical success was achieved in 55 patients (90.2%). Anatomical success was not achieved in six cases: one patient with concha hypertrophy and sinusitis, four patients with septal deviation, one patient with sinusitis alone, and one patient without any nasal pathology (Table 2). Importantly, there was no statistically significant difference in anatomical success between patients with and without nasal pathology (96.2%, 90.2%) (P=0.3).

**Table 2:** Anatomical success rates according to types of nasal pathology.

Nasal Pathology Type	Anatomical Success / Total Patients (n)	Success Rate (%)
Concha pathology+Sinusitis	22/23	95.7
Nasal septal deviation	17/21	81.0
Sinusitis	14/15	93.3
Deviated septum+Conchal pathology	2/2	100.0
No nasal pathology	25/26	96.2

Of the 87 patients who underwent paranasal sinus CT scanning, 80 had PACD, and five of these failed to achieve anatomical success. All these five patients had nasal pathologies. Of the seven patients with rE-DCR, two had no anatomical success and only one of them had nasal pathology. There was no statistically significant difference in anatomical success rates between patients with PACD and rE-DCR patients (P=0.09). Regression analysis showed that nasal pathologies had no significant effect on anatomical success (P=0.81; confidence interval (CI): 0.28-5.19; odds ratio (OR): 1.2).

## Discussion

In our study, external DCR with silicone tube intubation was performed in 87 epiphora patients admitted to the Eye Clinic between 2018 and 2023. The presence of nasal pathologies was retrospectively evaluated in all patients. Of the total cohort, 29.9% (n=26) demonstrated no detectable pathology, whereas 70.1% (n=61) exhibited various abnormalities, including inferior turbinate hypertrophy, sinus inflammation, septal deviation, or a combination of turbinate hypertrophy and septal deviation. Anatomical success was achieved in 55 out of 61 eyes (90%) with nasal pathology. There was no statistically significant difference in anatomical success between those with and without nasal pathology (P=0.3). Furthermore, there was also no statistically significant difference in anatomical success rates between patients with PACD and patients with rE-DCR (P=0.09). This may be due to the fact that all patients had silicone tube intubation for six months.

Although our study does not compare Ex-DCR with and without silicone intubation, the high anatomical success rate in patients with nasal pathologies may reflect a stabilizing effect of the silicone stent.

External dacryocystorhinostomy is recognized as a surgical method with a high success rate in the treatment of NLD [7]. However, the success of this procedure is closely tied not only to surgical technique and postoperative care but also to the patient's nasal anatomy and the presence of nasal pathologies. Anatomical abnormalities such as nasal septal deviation, conchal hypertrophy, and nasal polyps can present challenges during the surgery [16]. Particularly in cases of septal deviation, the asymmetry in the surgical area can complicate the application of surgical techniques. These abnormalities can also affect the proper patency of the anastomosis site during the postoperative period. In this context, the need for additional surgical interventions, such as septoplasty, has been a topic of frequent discussion [17,18].

Inflammatory conditions such as chronic rhinosinusitis can exacerbate mucosal inflammation, which in turn can negatively affect the healing process at the surgical site. This can increase the risk of postoperative stenosis and granulation tissue formation. Additionally, inflammation is known to delay healing in the tissues surrounding the ostium created during surgery and raise the risk of fibrosis. Therefore, controlling chronic inflammatory processes is critical for improving surgical outcomes [13]. Specifically, managing conditions like rhinosinusitis or nasal polyps with medical treatment plays a key role in enhancing surgical success [19].

Lin et al., in their study investigating the causes of failure in external and endoscopic DCR surgeries, found that adhesions involving the middle turbinate were particularly influential (57.1% versus 28.1%; P=0.04). They also reported that if the initial surgery was performed externally, the likelihood of needing septoplasty during revision surgery was higher (71.1% versus 15.6%; P=0.02) [14].

Yarmohammadi and colleagues performed preoperative paranasal sinus tomography on 50 cases of failed Ex-DCR before revision surgery. Sinus CT scans revealed at least one abnormality in 94% of cases. During revision endoscopy, nasal septum deviation (66%), scar formation (32%), ostium issues (28%), and sump syndrome (6%) were observed. The pathological and clinical findings showed a significant relationship between chronic inflammation and scar tissue as well as septal synechiae (P=0.001 and 0.008, respectively) [15].

Salih at al. reported that external DCR with intubation had a success rate of 85%, compared to 80% without intubation [20]. Numerous comparative studies have shown that the use of silicone intubation in primary DCR increases the success rate of the procedure compared to DCR without intubation [21]. There are many studies in the literature indicating that Ex-DCR with silicone tube intubation is more successful than external dacryocystorhinostomy without intubation [22,23].

In a meta-analysis of randomized controlled trials comparing silicone intubated and non-intubated DCR, a statistically significant improvement of 5% in success rates was observed with silicone intubation [24]. Another meta-analysis of randomized controlled trials found no statistical difference between non-intubated endoscopic, external, and transcanalicular laser DCR procedures. However, when nasal pathologies were present, endonasal DCR with silicone tube intubation was found to be a more suitable approach, while external DCR was preferred in the absence of nasal pathologies [25].

In our study, the anatomical success rate in 61 patients with nasal pathologies was 90% (55 patients), which was consistent with the literature. We believe that the reason for this could be the silicone tube intubation performed in dacryocystorhinostomy. The silicone tube likely maintained the ostium's patency and may not have been affected by nasal pathologies such as concha hypertrophy, sinusitis, and septal deviation. None of the patients experienced complications related to silicone intubation, such as punctal and canalicular lacerations, tube displacement, foreign body sensation, or conjunctival irritation, as the tubes were not removed before six months. Anatomical failure was observed in one patient with concha hypertrophy and sinusitis, four patients with septal deviation, one patient with only sinusitis, and one patient with no nasal pathology. There was no statistically significant difference in anatomical success between patients with nasal pathologies and those without (p>0.05).

Among the 80 patients with PACD, anatomical success was not achieved in five cases, all of which had nasal pathologies. In contrast, among seven patients with rE-DCR, anatomical success was not observed in two cases and only one of them had nasal pathology. There was no statistically significant difference in anatomic success between patients with either dacryocystitis (P>0.05).

This study has several limitations. First, its retrospective design and the relatively small number of patients with paranasal sinus pathologies limit the generalizability of the findings. Although nasal pathologies did not appear to significantly affect the anatomic success of Ex-DCR with silicone intubation in our cohort, prospective studies involving a larger number of patients with preoperative paranasal CT evaluation are warranted.

Secondly, although the mean follow-up duration was 2.45±2.14 years, anatomical success was defined solely based on lacrimal patency assessed one month after silicone tube removal. This early assessment provides an objective measure of surgical outcome but may not fully reflect long-term patency or functional success. In addition, although all patients underwent dacryoscintigraphy in the preoperative period, postoperative dacryoscintigraphy was not routinely performed. Anatomical success was instead evaluated using lacrimal irrigation, a widely accepted and practical method. However, postoperative dacryoscintigraphy could have provided further insight into functional outcomes, especially in cases of subclinical obstruction or persistent epiphora. This limitation was primarily due to institutional protocols and resource constraints, which reserve dacryoscintigraphy for symptomatic patients. Therefore, future prospective studies incorporating standardized long-term follow-up and both anatomical and functional assessments - including postoperative dacryoscintigraphy - are needed to better understand the durability and clinical effectiveness of Ex-DCR with silicone intubation.

Finally, limitations related to statistical analyses should be acknowledged. Due to the relatively small sample size, retrospective study design, and the low number of unsuccessful cases, multivariate analyses could not be performed. These factors limit the depth of statistical evaluation and may affect the robustness of the conclusions.

These findings suggest that bi-canalicular silicone intubation in Ex-DCR is an effective surgical approach for treating nasolacrimal duct obstruction, even in the presence of nasal pathologies [13-15]. However, such pathologies should be considered during surgical planning, although our data did not show a significant effect. Therefore, preoperative evaluation of nasal structures is essential and careful consideration of these conditions during surgical planning may enhance the overall outcome of Ex-DCR.

## Conclusions

In conclusion, our study suggests that Ex-DCR with bi-canalicular silicone intubation can achieve high short-term anatomical success rates in patients with nasolacrimal duct obstruction, irrespective of the presence of nasal pathologies. Although anatomical variations and inflammatory nasal conditions have the potential to influence surgical outcomes, the use of silicone stents may help maintain ostium patency during the postoperative healing phase. While the observed difference in anatomic success rates between patients with and without nasal pathologies was not statistically significant, these findings highlight the relevance of preoperative nasal evaluation. Nevertheless, the lack of long-term and functional outcome data limits the generalizability of the results, and further prospective studies are warranted to confirm these observations.
